# Description of the vaginal microbiota in nulliparous ewes during natural mating and pregnancy: preliminary signs of the male preputial microbiota modulation

**DOI:** 10.3389/fmicb.2023.1224910

**Published:** 2024-01-11

**Authors:** Marta Barba, Marion Toquet, Empar García-Roselló, Jesús Gomis, Juan J. Quereda, Pedro González-Torres, Belén Carbonetto, Ángel Gómez-Martín

**Affiliations:** ^1^Microbiological Agents Associated with Animal Reproduction (ProVaginBIO) Research Group, Departamento Producción y Sanidad Animal, Salud Pública Veterinaria y Ciencia y Tecnología de los Alimentos, Facultad de Veterinaria, Universidad Cardenal Herrera-CEU, CEU Universities, Carrer Tirant lo Blanc, Alfara del Patriarca, Valencia, Spain; ^2^Agrifood Research and Technology Centre of Aragon (CITA), Teruel, Spain; ^3^Research Group Intracellular Pathogens: Biology and Infection, Departamento Producción y Sanidad Animal, Salud Pública Veterinaria y Ciencia y Tecnología de los Alimentos, Facultad de Veterinaria, Universidad Cardenal Herrera-CEU, CEU Universities, Valencia, Spain; ^4^Microomics Systems S.L., Barcelona, Spain

**Keywords:** 16S metagenomics, reproductive microbiota, ovine, sheep, microbiome, pregnant, intravaginal sponge

## Abstract

The vaginal microbiota plays a key role in animals’ health. Understanding its diversity and composition and associated changes occurring through the reproductive cycle represents valuable knowledge to disclose the mechanisms leading to dysbiosis and eventually to infection. Even if the human vaginal microbiota has been thoroughly studied, scarce research has been conducted on the vaginal microbiota of livestock. In this study, 16S rRNA gene-based sequencing was performed on vaginal samples of ten nulliparous ewes at three different sampling points: before the estrus synchronization protocol (T0), at the time of estrus before mating (Testrus), and the day of the pregnancy diagnosis (Tpreg). Preputial samples from the three males collected pre and post-mating were also analyzed. Firmicutes, Proteobacteria, Bacteroidetes, and Actinobacteria were the most abundant phyla in vaginal samples. The most abundant genera were *Porphyromonas*, *Anaerococcus*, and *Peptinophilius*. Vaginal microbiota biodiversity decreased during pregnancy. Tenericutes (*Ureaplasma* spp.) increased significantly at Tpreg in both pregnant and non-pregnant ewes. Differences were observed between pregnant and non-pregnant ewes at Tpreg where pregnant ewes had a significantly higher abundance of *Actinobacillus* spp. and *Ureaplasma* spp. Ewes that were diagnosed with pregnancy at Tpreg showed a decreased abundance of gram-negative bacteria such as Bacteroidales, Campylobacterales, and Enterobacteriales. In addition, a significant decrease in the relative abundances of genera within Firmicutes, such as *Alloicoccus* (Lactobacillales), *Atopostipes* (Lactobacillales), and an uncultured bacteria W5053 from Family XI (Firmicutes, Clostridiales) was observed in non-pregnant ewes at Tpreg. The four most abundant phyla in the rams’ prepuce were the same as in the ewes’ vagina. The most abundant genus was *Corynebacterium*. No major differences were observed in the ram’s preputial microbiota between pre and post-mating samples. Nevertheless, the differences in the taxonomic composition of ewes’ vaginal microbiota between Testrus and Tpreg could be explained by the exposure to the preputial microbiota. This study offers new insights into the effects of several key steps of the ewe’s reproductive cycle such as estrus-synchronization protocol, mating, and pregnancy on ovine vaginal microbiota. The knowledge of the microbiota dynamics during the reproductive cycle can help improve the reproductive outcomes of dams by identifying biomarkers and putative probiotics.

## 1 Introduction

Vaginal microbiota plays an important role in the inhibition of reproductive pathogenic microorganisms (Foschi et al., 2017). Healthy human vaginal microbiota has been well described and it is known to be characterized by the predominance of Lactobacillus spp. (Rönnqvist et al., 2006). Nevertheless, asymptomatic, healthy women, with a vaginal microbiota where *Lactobacillus* spp. are not dominant and where the vaginal pH is >5, do exist (Ma et al., 2012). In other mammals, such as ruminants, *Lactobacillus* spp. do not dominate vaginal microbiota (Wang et al., 2013; Swartz et al., 2014; Laguardia-Nascimento et al., 2015; Serrano et al., 2020; Quereda et al., 2020a). The lack of lactobacilli in the vaginal microbiota of livestock species makes its pH near neutral, opposed to the low pH (3.5–4.5) in humans (Swartz et al., 2014). These differences between human and livestock species imply that findings on the influence of the microbiota on fertility in women cannot be extrapolated to livestock species (Messman et al., 2020). As of this date, the composition and ecology of the vaginal microbiota of small ruminants have been scarcely described (Swartz et al., 2014; Serrano et al., 2020; Koester et al., 2021; Reinoso-Peláez et al., 2023) as most studies have focused on the isolation of specific pathogens related to vaginal infections but have not studied the composition of the whole microbial community (Manes et al., 2013; Vasconcelos et al., 2016; Bragança et al., 2017).

Culture-dependent methods allow studying only a small percentage of the microbiota (Serrano et al., 2020). Nevertheless, culture-independent methods, such as metagenomic analysis allow the characterization of nearly the complete microbiota composition. This method has already been used to study the microbiota of other species such as cows and mares during different reproductive stages (Bicalho et al., 2017; Barba et al., 2020). Knowledge of the dynamics of the vaginal microbiota will aid in better understanding dysbiosis, such as vaginosis induced by estrus-synchronization devices, and consequently the physiopathology of diseases such as vaginitis in small ruminants. These intravaginal devices are frequently used for estrus synchronization in sheep such as CIDR^®^ (based on silicon impregnated in natural progesterone) or polyurethane vaginal sponges (with synthetic progestagen). Histological, cytological, and microbiological changes in vaginal samples after sponge removal have been well described (Gatti et al., 2011; Manes et al., 2015; Martinez-Ros et al., 2018; Quereda et al., 2020b). These changes can comprise abnormal vaginal discharge and adhesions, which can negatively affect fertility (Scudamore, 1988). The effects of synchronization devices on the vaginal microbiota of ewes have been addressed, showing the proliferation of *Enterobacteriaceae* (Quereda et al., 2020b), and changes in bacterial diversity (Manes et al., 2015). Moreover, antibiotics are usually used to prevent vaginal infections caused after the application of the devices and can produce changes in the bacterial population (Serrano et al., 2020). The use of sponges with antibiotics in goats has been reported as a main risk for the presence of antibiotic residues in milk, discouraging its use to control vaginosis/dysbiosis caused by the synchronization protocol (Romero et al., 2016). Hence, getting broader insight on the dynamics of the vaginal microbiota is essential. A metagenomics-based approach to study these dynamics could aid in finding potential markers for dysbiosis or even potential probiotic bacteria that could prevent the need for antibiotics (Otero et al., 2006; Serrano et al., 2020).

Not only sponge devices and antibiotics are factors that can affect the vaginal microbiota, but the male preputial microbiota could also have effects on the vaginal microbiota and *vice-versa* during mating. Preputial and semen microbiota in domestic ruminants have been described in bulls (Wickware et al., 2020; Cojkic et al., 2021) and bucks (Mocé et al., 2022). Moreover, the presence of *Actinobacillus seminis* and *Histophilus somni*, known to be related to infertility, has been observed in the preputial microbiota of service rams from a herd with low artificial insemination success (Serrano et al., 2020).

The composition and stability of the vaginal microbiota of normal pregnant women are different from that of non-pregnant women (Ma et al., 2012; Romero et al., 2014; Freitas et al., 2017). Moreover, dysbiosis in vaginal microbiota composition is associated with high-risk pregnancy (Freitas et al., 2017; Tabatabaei et al., 2019; Di Simone et al., 2020). These affirmations are not so clear for livestock animals. Chen et al. (2020) have not observed differences in vaginal microbiota diversity between pregnant and non-pregnant cattle after artificial insemination. On the contrary, reduced bacterial species diversity in pregnant *Bos indicus* cattle compared to non-pregnant animals has been reported (Laguardia-Nascimento et al., 2015). Up to date, only one study using metagenomics on vaginal microbiota changes during pregnancy in multiparous ewes is available (Koester et al., 2021) although some studies have described the differences in microbiota between pregnant and non-pregnant ewes before the artificial insemination (Serrano et al., 2020; Reinoso-Peláez et al., 2023). Nevertheless, no report has been found on the study of the vaginal microbiota in nulliparous ewes.

This work is based on the hypothesis that estrus synchronization with sponges and natural mating affects ovine vaginal microbiota. Changes can be positive or detrimental, based on the output of natural mating/pregnancy. The use of nulliparous females would reduce interferences typical of multiparous females. We used a 16S rRNA gene-based sequencing approach to describe the diversity and composition of vaginal microbiota in nulliparous healthy ewes during mating. The main objective was to compare the vaginal microbiota before and after the removal of the estrus synchronization sponge and at pregnancy diagnosis. Secondly, we aimed to compare the male preputial microbiota before and after mating.

## 2 Materials and methods

### 2.1 Ethics statement

All experimental procedures were approved by the University CEU Cardenal Herrera Care and Use Committee for Livestock and by the Spanish Regional Government Generalitat Valenciana (Animal use protocol 2018/VSC/PEA/0183). Ewes were cared for in accordance with the guidelines of the Spanish Policy for Animal Protection (RD53/2013) which complies with the European Union Directive 2010/63/UE about the protection of animals used for research.

### 2.2 Animals and experimental design

The study was conducted at the University CEU Cardenal Herrera Research Farm (Náquera, Valencia, Spain latitude 39°N), that includes a total of 42 sheep, during the non-reproductive season from May to November. Ten 1 year-old female, which represented all the rearing nulliparous ewes at the time, and three 2 years-old male sheep (Romanov X Île de France) were included. The herd of this study was officially brucellosis-free. All the ewes and rams of the herd were also negative to the annual surveillance of *Mycoplasma agalactiae*, *Coxiella burnetti* and *Listeria* spp. Physical examination was performed on all animals and no signs of disease were observed in the animal population. The animals’ diet was hay and branch alfalfa and variations in the same were not carried out. All animals used in this study were born on the experimental farm. Movements in or out of animals were absent in the herd during the study period, as well as treatments or prophylactic regimens. All the females and males had not been used for reproduction before the experiment, therefore the animals had not suffered any previous manipulation which could have altered their reproductive microbiota.

To investigate the effect of sponge introduction, natural mating, and pregnancy in vaginal microbiota, vaginal swabs from synchronized ewes were obtained from the following sampling-points:

- T0 (the synchronization day, before sponge insertion).
- Testrus (two days after sponge removal).
- Tpreg (the day of pregnancy diagnosis, 50 days after sponge removal).

To investigate the effect of natural mating in preputial microbiota in males, preputial swabs were obtained on the following days:

- Tpre (previous to natural mating, the day of introduction with the ewes).
- Tpost (after natural mating, the day of removal from the ewes).

Ewes were treated using the protocols established in commercial farms. One intravaginal progestagen-impregnated sponge (20 mg fluorogestone acetate, FGA, Chronogest^®^; MSD Animal Health, Madrid, Spain) was inserted for 14 days. At sponge removal, animals received one intramuscular injection of 350 IU of equine chorionic gonadotropin (eCG) (Foligon^®^, MSD Animal Health, Madrid, Spain). Previously to the application of the sponge, the vulva area was disinfected, and all the material used for the application of the sponge was disinfected between animals. After sampling the ewes for Testrus, the three males were introduced and allowed to remain with the ewes for four days. In order to reproduce the random natural mating that occurs routinely in the herds, we did not intervene in it by carrying out selective mating. The males were with the nulliparous ewes in an enclosure of 1,000 m^2^ to avoid natural mating only by dominant males. It was also ruled out to include the rams with the highest hierarchical scale in the flock in the study. The three males were watched actively mating. Pregnancy was diagnosed by transabdominal ultrasonographic examination (NanoMaxx, Sonosite, Bothell, WA, United States) 50 days after sponge removal and confirmed at lambing.

After pregnancy confirmation, experimental groups were subdivided into pregnant (P, *n* = 6) and non-pregnant (NP, *n* = 4) ewes, in order to investigate if differences in vaginal microbiota existed between P and NP groups at any sampling time point. Therefore, ewes were divided into six experimental groups: T0_P, T0_NP, Testrus_P, Testrus_NP, Tpreg_P, and Tpreg_NP.

### 2.3 Vaginal sampling procedures

At T0, Testrus, and Tpreg, vaginal swabs were collected from each of the ten ewes. Contamination was prevented by thoroughly cleaning with water and neutral soap the vulvar area. Care was taken to avoid the moment of defecation and urination while sampling. Then the vulva was opened by one operator and the second operator opened the sterile DNA-free cotton swab (Deltalab^®^-ref. 300,263) and introduced it into the vaginal tract via the opened vulva, without touching the vulva or the external urethral orifice until the cranial vagina. Samples were obtained by gently swabbing the vaginal wall for 30 s. Then it was extracted carefully via the same methodology, avoiding contact with the vulva, and it was introduced in the transport tubes. The methodology was followed as previously described (Nugeyre et al., 2019; Quereda et al., 2020a). Swabs were quickly stored at −80°C.

### 2.4 Preputial sampling procedures

At Tpre and Tpost, preputial swabs were collected from each of the three males while they were in supine position with their four legs and head immobilized. Contamination was prevented by cleaning with water and neutral soap the external skin of the preputial area. Then the preputial orifice was opened by one operator and the second operator opened the sterile DNA-free cotton swab (Deltalab^®^-ref. 300,263) sterile and introduced it inside the preputial sac. After swabbing for 30 s, it was extracted carefully avoiding contact with the external skin, and it was introduced in the transport tubes. The swabs were not grossly contaminated with dirt or debris. The methodology was followed as previously described (Wickware et al., 2020). Swabs were quickly stored at −80°C.

### 2.5 Library preparation and sequencing

DNA was extracted from swab samples using the DNeasy PowerLyzer PowerSoil Kit (Qiagen, Hilden, Germany) following the manufacturer’s instructions. The extraction tubes were agitated using Tissue lyser II (Qiagen, Hilden, Germany) at 30 Hz/s for 10 min at 4°C. Mock community DNA was included as positive control for library preparation (Zymobiomics Microbial Community DNA, Catalog Nos. D6305, ZymoResearch, Irvine, CA, United States). Samples were amplified using primers specific to the V3–V4 regions of the 16S rRNA DNA (V3–V4-Forward 5′-TCGTCGGCAG CGTC AGATGTGTA TAAGAGACAGCCTACGGGNGGCWGCAG-3′, V3–V4 Reverse 5′GTCTCGTGGGCTCGGAG ATGTGTATAAGAGAC AGGACTACHVGGGTATCTAATCC-3′).

The PCR was performed in 10 μL final volume with 0.2 μM primer concentration. The PCR included: 3 min at 95°C (initial denaturation) followed by 25 cycles: 30 s at 95°C, 30 s at 55°C, and 30 s at 72°C, and a final elongation step of 5 min at 72°C. PCR products were purified using AMPure XP beads (Beckman Coulter, Nyon, Switzerland) with a 0.9× ratio according to the manufacturer’s instructions. PCR products were eluted from the magnetic beads with 32 μL of Milli-Q water and 30 μL of the eluate were transferred to a fresh 96-well plate. The above-described primers contain overhangs allowing the addition of full-length Nextera barcoded adapters for multiplex sequencing in a second PCR step, resulting in sequencing-ready libraries with approximately 450 bp insert sizes. In brief, 5 μL of the first PCR purified product were used as the template for a second PCR with Nextera XT v2 adaptor primers in a final volume of 30 μL using the same PCR mix and thermal profile as for the first PCR but with only eight cycles. 25 μL of the second PCR product were purified with SequalPrep normalization kit (Invitrogen, ThermoFisher Scientific, Waltham, MA, United States), according to the manufacturer’s protocol. Libraries were eluted in 20 μL final volume and pooled for sequencing. The final pool was quantified by qPCR using Kapa library quantification kit for Illumina Platforms (Kapa Biosystems, Sigma Aldrich, Saint Louis, MO, United States) on an ABI 7900HT real-time cycler (Applied Biosystems, ThermoFisher Scientific, Waltham, MA, United States). Sequencing was performed using Illumina MiSeq with 2 × 300 bp reads using v3 chemistry with a loading concentration of 10 pM. In all cases, 15% of PhIX control libraries were used to increase the diversity of the sequenced sample. Negative controls included sample collection buffer, DNA extraction, and PCR amplification steps, PRC products after both PCR steps were visualized using an electrophoresis gel (1.5% agarose) with SYBR Safe (Applied Biosystems, ThermoFisher Scientific, Waltham, MA, United States). No visible bands were observed. Amplification of the mock community standard was expected, 450 bp-size amplicons were obtained.

### 2.6 Amplicon sequences processing and analysis

Raw demultiplexed forward and reverse reads were processed using the following methods and pipelines as implemented in QIIME2 version 2019.4 with default parameters unless stated (Bolyen et al., 2019). DADA2 was used for quality filtering, denoising, pair-end merging, and amplicon sequence variant calling using qiime dada2 denoise-paired method (Callahan et al., 2016). Q20 was used as quality threshold to define read sizes for trimming before merging (parameters: --p-trunc-len-f and --p-trunc-len-r). Reads were truncated at the position when the 75th percentile Phred score felt below Q20: 300 bp for forward reads and 242 bp for reverse reads. After quality filtering steps, the average sample size was 33,144.8 reads (min: 13,680 reads, max: 58,336 reads). Amplicon Sequence Variants (ASVs) were aligned using the qiime alignment mafft method (Katoh and Standley, 2013). The alignment was used to create a tree and to calculate phylogenetic relations between ASVs using qiime2 phylogeny fasttree method (Price et al., 2009). ASV tables were subsampled without replacement in order to even sample sizes for diversity analysis using qiime diversity core-metrics-phylogenetic pipeline. The smallest sample size was chosen for subsampling (Lozupone and Knight, 2005). Unweighted and weighted Unifrac distances were calculated to compare community structure. Alpha diversity metrics: observed ASVS (i.e. richness), Pilou’s evenness index, and Shannon index were calculated. Taxonomic assignment of ASVs was performed using a Bayesian Classifier trained with Silva database (i.e., 99% ASVs database) using the qiime feature-classifier classify-sklearn method (Abraham et al., 2014). Since the swab samples could contain vaginal tissue cells, ASVs were filtered to discard contaminant Eukariota DNA-derived amplicons using Blast against the mentioned database with a 90% identity cutoff. The sample from ewe 8 at Testrus was discarded since the sample did not contain sufficient DNA. The taxonomic profile of the mock community control matched the expected bacterial profile.

### 2.7 Statistical analysis

Differential abundance of taxa was tested using ANCOM and Kruskal Wallis non-parametric test on relative abundance of taxa (total sum scale) (Mandal et al., 2015). After Kruskal Wallis, Conover’s test with FDR Benjamini–Hochberg correction was added for pairwise comparison. Alpha diversity comparisons were performed using Kruskal–Wallis non-parametric test. Beta diversity distance matrices were used to calculate principal coordinates analysis (PCoA) and to make ordination plots using R software package version 3.6.0^1^. The significance of groups in community structure was tested using Permanova. Permdisp test was used to identify location vs. dispersion effects (Anderson and Walsh, 2013). Significant threshold was set at *p* < 0.05. BiodiversityR version 2.11-1, PMCMR version 4.3, RVAideMemoire version 0.9-7 and vegan version 2.5-5 packages were used to generate PCoA plots and to perform Permanova, Permdisp and pair-wise Permanova tests. Kruskal Wallis rank sum test was used to compare Firmicutes/Bacteriodetes ratio between pregnancy and nonpregnancy groups at the different sampling times.

## 3 Results

### 3.1 Ovine vaginal microbiota

A total of 3,418,900 pair-end reads were obtained. After quality filtering, trimming, and denoising steps, 1,890,939 reads remained. Paired-end reads were merged and after chimera removal, 927,344 merged reads were used for phylotype calling with DADA2 (Callahan et al., 2016). Finally, 3,166 phylotypes were detected. Singletones and doubletones were removed before diversity analysis. The data have been deposited and can be found in the European Nucleotide Archive (ENA) (European Molecular Biology Laboratory, European Bioinformatics Institute (EMBL-EBI)): https://www.ebi.ac.uk/ena/browser/home, with the study ID PRJEB63647 (ERP148797).

#### 3.1.1 Vaginal community richness, evenness, and diversity decreases at time of pregnancy diagnosis, especially in non-pregnant ewes

Rarefaction curves showed that the achieved sequencing depth and subsampling size were enough to observe the diversity present in the samples since a plateau was reached (Supplementary Figure S1).

**Figure 1:**
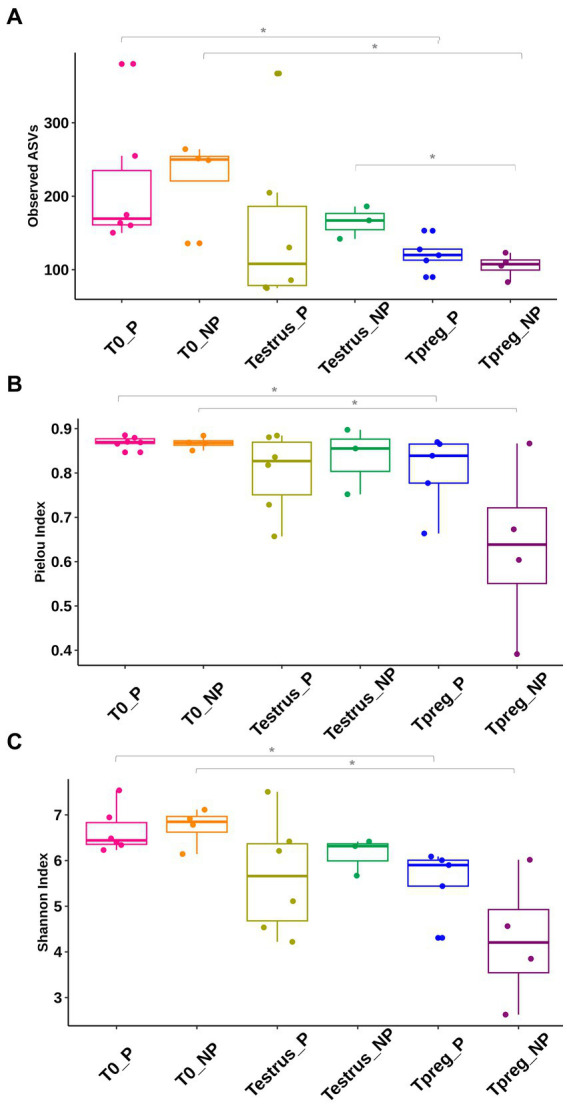
Comparison of vaginal microbial diversity: Community richness **(A)**, Pielou’s Evenness **(B)**, Shannon diversity index **(C)**, of 10 ewes at T0 (the day of the sponge insertion), Testrus (2 days after sponge removal) and Tpreg (the day of pregnancy diagnosis, 50 days after sponge removal) divided in pregnant (P) and nonpregnant (NP) groups. Each sample is represented as a dot. Boxplot includes median, quartile and confidente interval representation. *p* < 0.05 statistical significance is expressed with *.

Higher values were observed in all three alpha diversity indices (community richness, Pielou’s evenness and Shannon diversity index) in T0_P compared to Tpreg_P (*p* < 0.05) and in T0_NP compared to Tpreg_NP (*p* < 0.05) (Figure 1; Supplementary Table S1). Richness (observed ASVs) was significantly higher in the microbiota of Testrus_NP compared to Tpreg_NP (*p* < 0.05) (Figure 1; Supplementary Table S1). No significant differences were observed for alpha diversity indexes between pregnant and non-pregnant ewes at the same sampling time point (Figure 1; Supplementary Table S1).

#### 3.1.2 Microbial community structure changes after sponge removal and at time of pregnancy diagnosis in pregnant ewes

**Figure 2:**
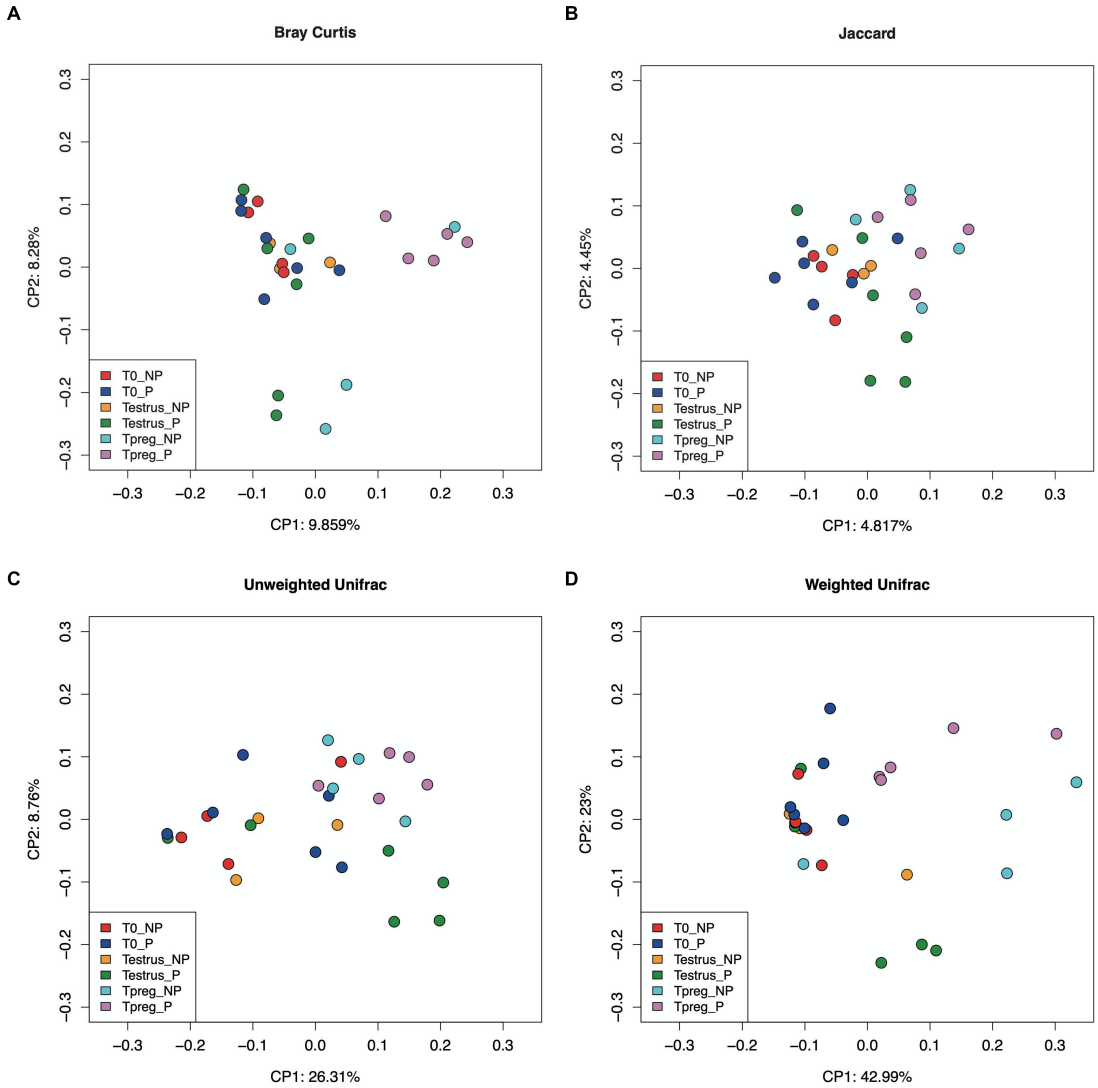
Beta diversity analysis in ordination plot (PCoA) based Bray–Curtis **(A)**, Jaccard **(B)**, Unweighted Unifrac **(C)**, and Weighted Unifrac **(D)** distances, of 10 of 10 ewes at T0 (the day of the sponge insertion), Testrus (two days after sponge removal) and Tpreg (the day of pregnancy diagnosis, 50 days after sponge removal) divided in pregnant (P) and nonpregnant (NP) groups. *p* < 0.05 statistical significance is expressed with *.

Differences were observed in the microbial community structure between Tpreg_P and T0_P samples and between Tpreg_P and Testrus_P (Permanova *p* < 0.05, Supplementary Table S2), for unweighted Unifrac (0.030), weighted Unifrac (0.020), Jaccard (0.015), and Bray Curtis (0.030) distances. The PCoA of individual samples for the distances mentioned above are shown in Figure 2. No significant differences were observed for the calculated distances between the other experimental groups and sampling time points (Supplementary Table S2).

#### 3.1.3 Firmicutes was the most predominant phylum in vaginal samples from nulliparous ewes, except in non-pregnant ewes at time of pregnancy diagnosis

The V3–V4 region of the 16S rRNA gene used in this study allowed the detection of both Bacterial and Archaeal communities. Bacteria and Archaea were detected in 100% (29/29) and 65.5% (19/29) of vaginal samples, respectively.

**Figure 3:**
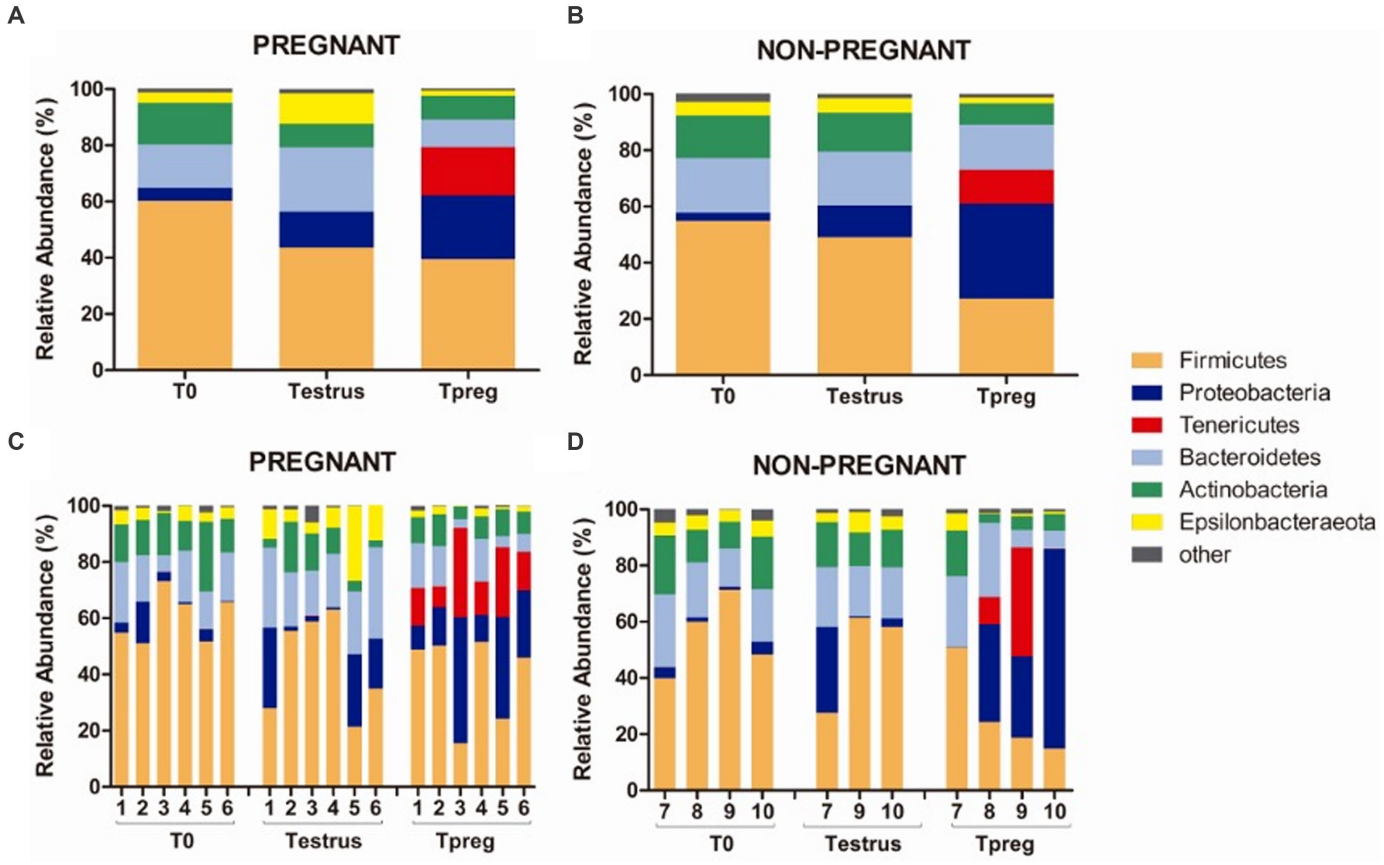
Phylum taxonomic relative abundance from ovine vaginal samples. Only taxa with a mean relative abundance >1.5% at any sampling time point are represented. Mean relative abundance in pregnant **(A)** and non-pregnant ewes **(B)** at T0 (the day of the sponge insertion), Testrus (two days after sponge removal) and Tpreg (the day of pregnancy diagnosis, 50 days after sponge removal). Relative abundance in individual samples in pregnant **(C)** and non-pregnant **(D)** ewes at the same sampling points (each column represents an individual sample).

Twenty bacterial phyla were observed. The most abundant bacterial phyla were Firmicutes, Bacteroidetes, and Proteobacteria. Firmicutes was the most abundant phylum both in P and NP ewes at all sampling points (ranging from 39.42% to 60.28% mean relative abundance, Figure 3), except for Tpreg_NP, where the most abundant phylum was Proteobacteria (33.74%) (Figure 3). The following abundant phyla were Actinobacteria, Tenericutes, and Epsilonbacteraeota. One archeal phylum (Euryarchaeota) was identified.

#### 3.1.4 Clostridiales was the most abundant order in vaginal samples from nulliparous ewes, except in non-pregnant ewes at time of pregnancy diagnosis

**Figure 4:**
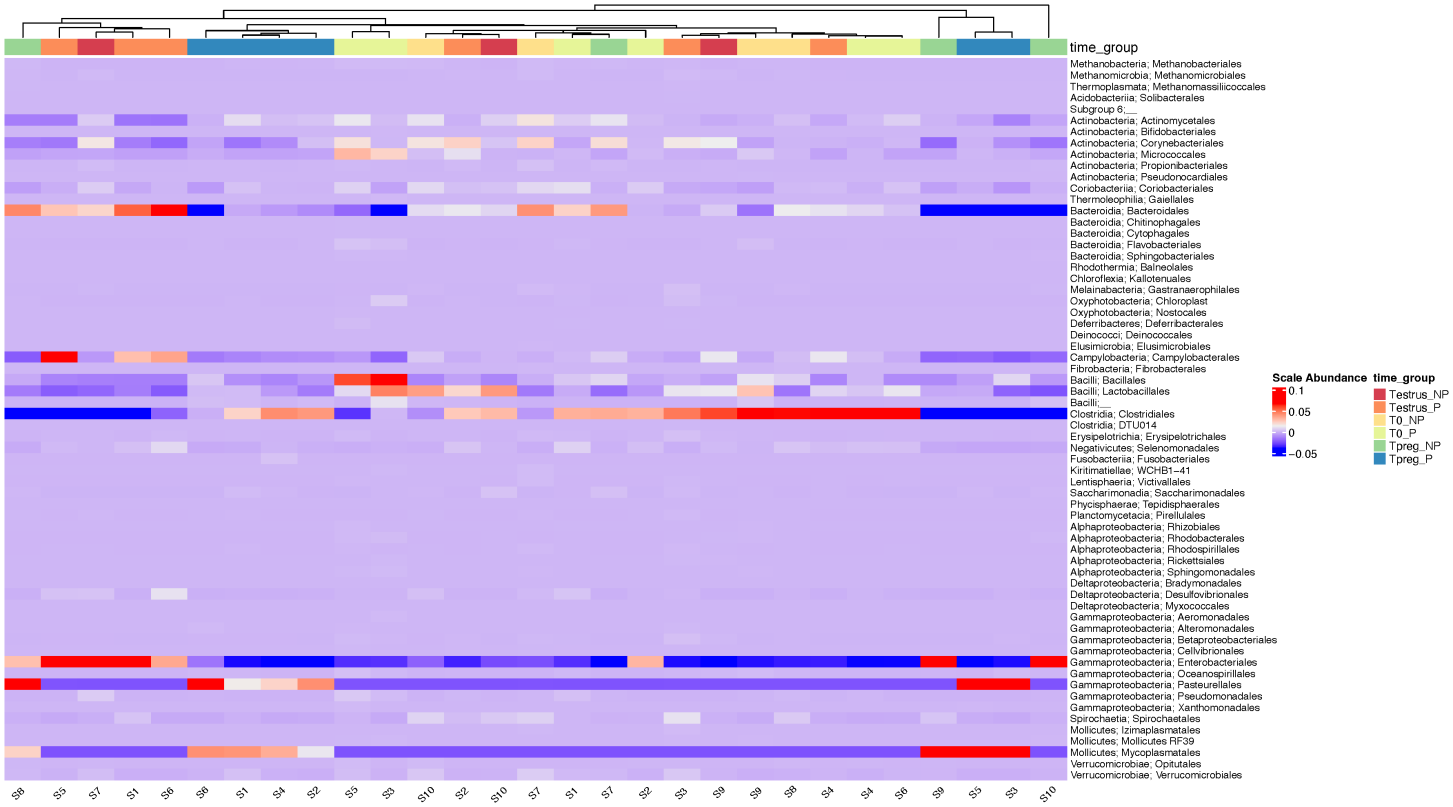
Heatmap of the relative abundance at the order level for each ovine individual vaginal sample at the different sampling points: T0 (the day of the sponge insertion), Testrus (two days after sponge removal) and Tpreg (the day of pregnancy diagnosis, 50 days after sponge removal) in both pregnant (P) and non-pregnant (NP) ewes. Samples are clustered based on the similarities of their microbial composition.

The most abundant order in every experimental group was Clostridiales, except for Tpreg_NP, which was Enterobacteriales. Bacteroidales was the second most abundant order at T0 and Testrus in both P and NP groups (Supplementary Figure S2). The group Tpreg_P was the one that showed the most similarities in terms of the taxonomic composition, at the order level, of its individual samples (Figure 4).

#### 3.1.5 Porphyromonas was the most abundant genus in vaginal samples from nulliparous ewes both in pregnant and non-pregnant ewes

**Figure 5:**
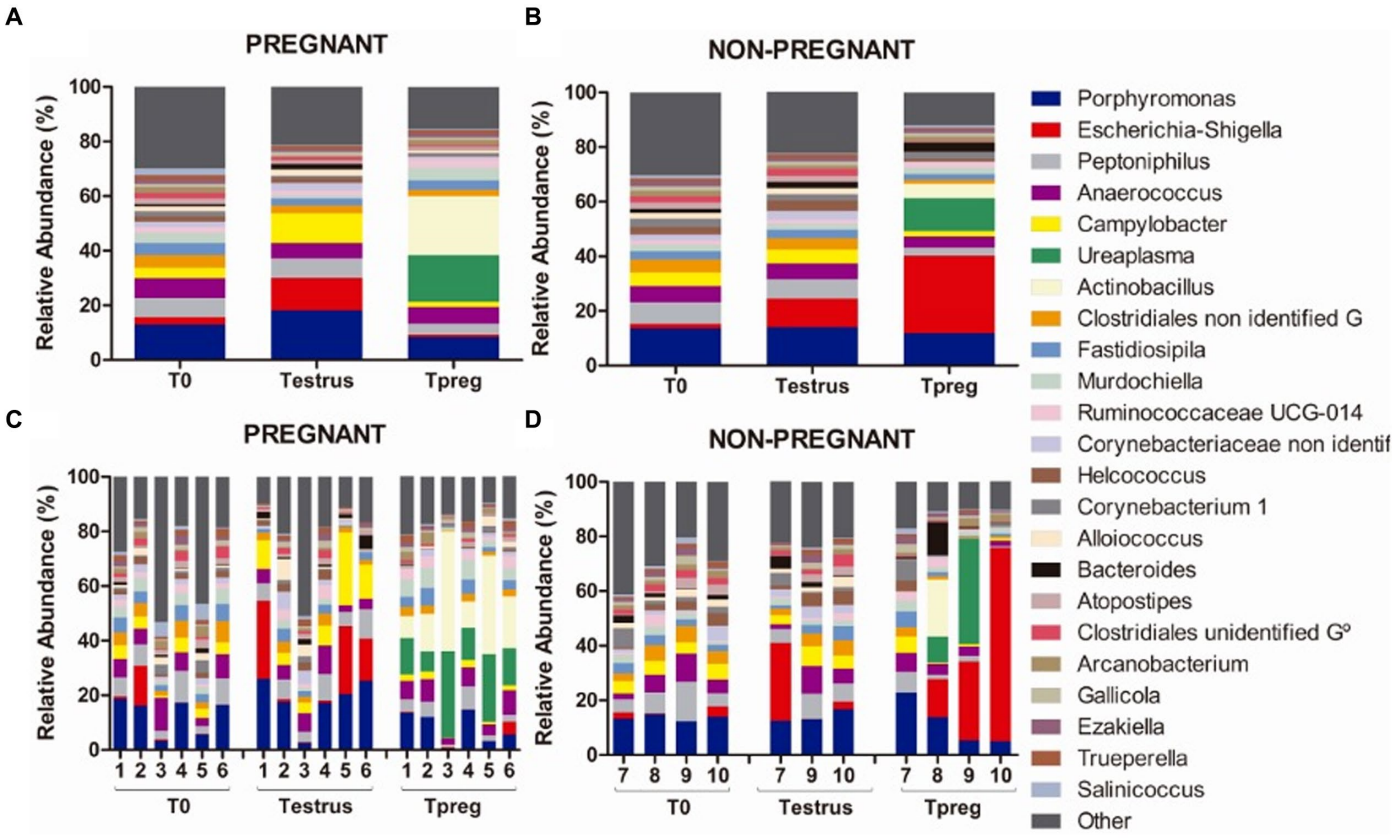
Genus taxonomic relative abundance from ovine vaginal samples. Only taxa with a mean relative abundance >1.5% at any sampling time point are represented. Mean relative abundance in pregnant **(A)** and non-pregnant ewes **(B)** at different sampling points: T0 (the day of the sponge insertion), Testrus (two days after sponge removal) and Tpreg (the day of pregnancy diagnosis, 50 days after sponge removal). Relative abundance in individual samples in pregnant **(C)** and non-pregnant **(D)** ewes at the same sampling points (each column represents an individual sample).

Results of most abundant genera (>1.5% relative abundance) are represented in Supplementary Table S3. The most abundant (>5% relative abundance) genera at every time point in both P and NP groups were *Porphyromonas* (Bacteroidetes, Bacteroidales), *Peptoniphilus* (Firmicutes, Clostridiales), and *Anaerococcus* (Firmicutes, Clostridiales) (Figure 5).

At the species level, six species with >1.5% relative abundance were identified from the most abundant genus: *Porphyromonas* sp. HMSC077F02, *Porphyromonas* sp. 2007b, *Porphyromonadaceae* bacterium FC4, *Porphyromonas* sp.2018, uncultured *Porphyromonadaceae* bacterium, and unidentified *Porphyromonas*.

#### 3.1.6 Pregnant ewes showed a decrease of Bacteroidales, Campylobacterales, and Enterobacterales at the time of gestation, which was not observed in non-pregnant ewes

At Testrus, in addition to the three most abundant genera, other genera containing pathogenic species were abundant including *Escherichia-Shigella* (Proteobacteria, Enterobacteriales), *Campylobacter* (Epsilonbacteraeota, Campylobacterales) and *Bacteroides* (Bacteroidetes, Bacteroidales) both in NP and P groups (Figure 5).

**Figure 6:**
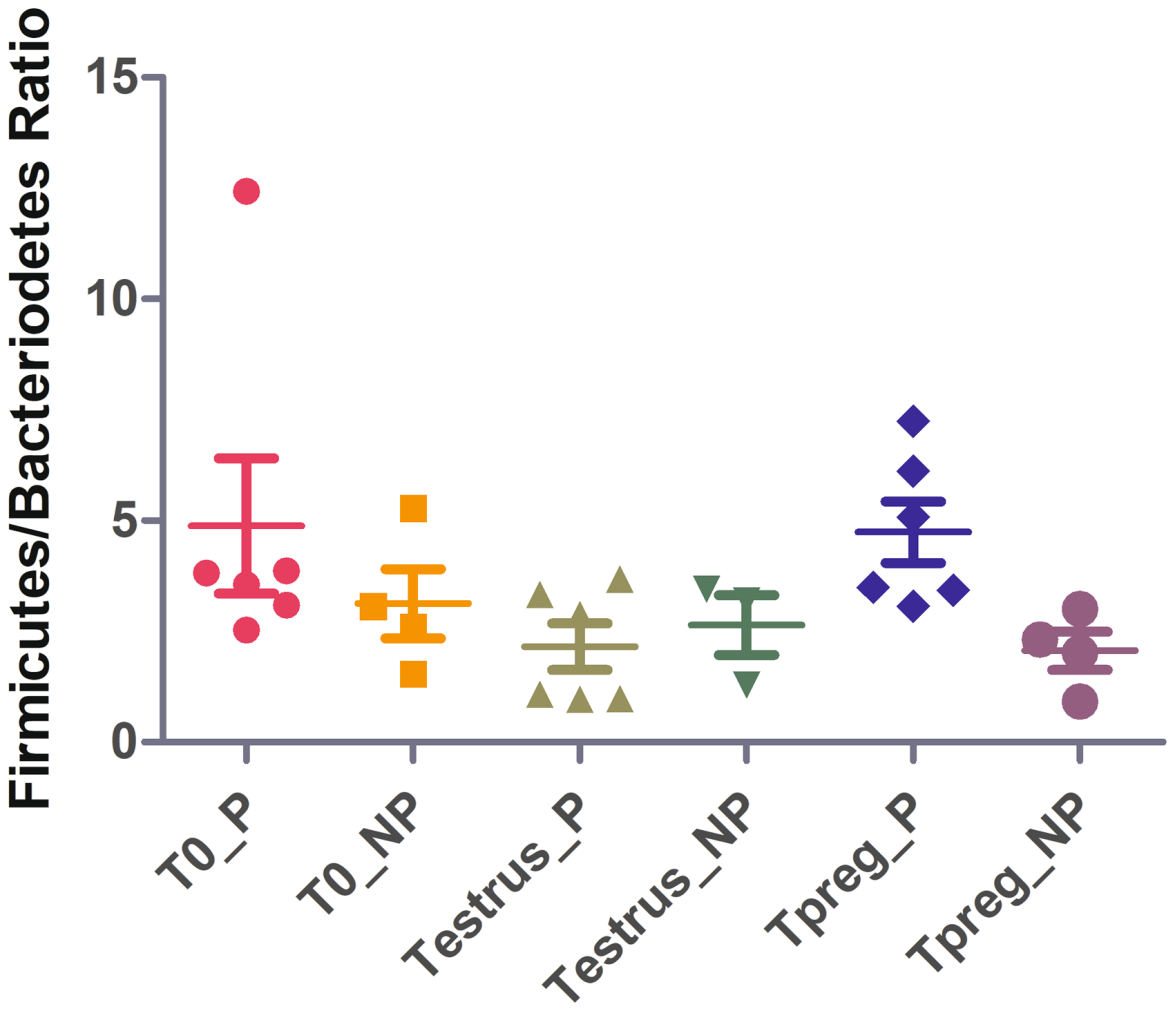
Strip chart showing median and interquartile range for Firmicutes/Bacteriodetes ratio values of ovine vaginal samples at the different sampling points: T0 (the day of the sponge insertion), Testrus (two days after sponge removal) and Tpreg (the day of pregnancy diagnosis, 50 days after sponge removal) in both pregnant (P) and non-pregnant (NP) ewes. *p* < 0.05 statistical significance by Kruskal Wallis rank sum test is expressed with *.

At Tpreg, a decrease in abundance of these three taxa was observed only in pregnant ewes at the phylum, order and genus level. Bacteroidetes, Bacteroidales and *Bacteroides* were significantly less abundant at Tpreg than Testrus in P group (*p* < 0.05). The Firmicutes/Bacteroidetes ratio was significantly lower (*p* < 0.01) in NP group (2.06) compared to P group (4.74) at Tpreg (Figure 6).

Epsilonbacteriota (*p* < 0.05), Campylobacterales (*p* = 0.01), and *Campylobacter* (*p* < 0.05) were significantly less abundant at Tpreg than Testrus in P group. *Campylobacter* species identified were *Campylobacter corcagiensis* and an uncultured *Campylobacter* sp.

*Escherichia-Shigella* was the most abundant genus only in NP ewes.

#### 3.1.7 Ureaplasma and Actinobacillus were more abundant in vaginal samples at time of pregnancy diagnosis, especially in pregnant ewes

At Tpreg *Actinobacillus* (Proteobacteria, Pasteurellales) and *Ureaplasma* (Tenericutes, Mycoplasmatales) were the most abundant genera in P ewes and among the most abundant in NP ewes (Figure 5).

**Figure 7:**
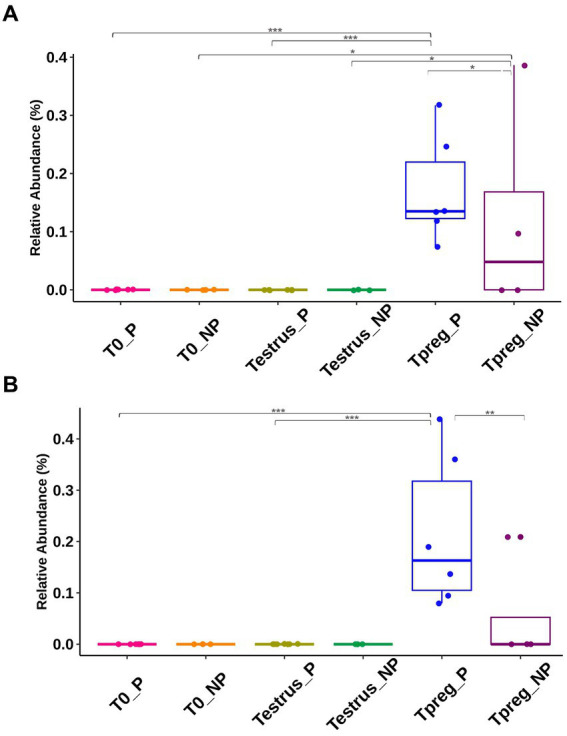
Taxonomic relative abundance of Genera Ureaplasma **(A)** and Actinobacillus **(B)** in ovine vaginal samples form pregnant (P) and non-pregnant ewes (NP) at different sampling points: T0 (the day of the sponge insertion), Testrus (two days after sponge removal) and Tpreg (the day of pregnancy diagnosis, 50 days after sponge removal). Kruskal–Wallis significance is expressed as * for *p* < 0.05, ** for *p* < 0.01, and *** for *p* < 0.001.

Significant differences in taxonomic composition were observed both at the phylum, order and genus level. Tenericutes was significantly more abundant in Tpreg_P compared to T0_P (*p* < 0.01) and Testrus_P (*p* < 0.01) and compared to Testrus_NP (*p* < 0.05) (Supplementary Table S3). Pasteurellales and Mycoplasmatales were abundant orders only at Tpreg (Supplementary Figure S1), both being significantly more abundant in P group compared to NP group (*p* < 0.01 and *p* = 0.01 respectively). Pasteurellales were significantly more abundant at Tpreg compared to T0 (*p* < 0.001) and Testrus (*p* < 0.01) in P group. Mycoplasmatales were more abundant at Tpreg compared to the other two sampling points both in P (*p* < 0.001) and NP groups (*p* = 0.01). Regarding *Ureaplasma*, it was more abundant in Tpreg compared to T0 and Testrus in both P and NP groups, and it was more abundant Tpreg_P compared to Tpreg_NP (Figure 7A; Supplementary Table S3). Furthermore, *Ureaplasma* was present in all ewes of P group, but only in two NP ewes (Figure 5). Regarding *Actinobacillus*, it was more abundant only in Tpreg_P compared to T0_P and Testrus_P, and it was more abundant in P compared to NP group at Tpreg (Figure 7B; Supplementary Table S3).

At the species level, the only species with >1.5% relative abundance identified within *Actinobacillus* genus was *Actinobacillus seminis*, and within *Ureaplasma* genus was *Ureaplasma* sp. USP128. Regarding the rest of the abundant genera, the most abundant species corresponded to uncultured bacteria, or it was not possible to identify the species.

#### 3.1.8 Non-pregnant ewes showed less abundance of some genera from Lactobacillales at time of pregnancy diagnosis

In NP ewes, other abundant genera that showed significant differences were: *Alloiococcus* (Firmicutes, Lactobacillales), *Atopostipes*, (Firmicutes, Lactobacillales), and uncultured bacteria W5053 from Family XI (Firmicutes, Clostridiales), which were less abundant at Tpreg than T0 and Testrus (Supplementary Table S3).

Regarding *Lactobacillus* spp., only two species were identified in P group and presented low relative abundance. *Lactobacillus mucosae* was observed in one animal at Testrus (0.06%) and an unidentified *Lactobacillus* sp. was observed in two different individuals at Tpreg (0.39% and 0.02%). No *Lactobacillus* spp. were identified in the NP group.

### 3.2 Ram preputial microbiota

A total of 840,003 pair-end reads were obtained. After quality filtering, trimming, and denoising steps, 43,0.862 reads remained. Paired-end reads were merged and after chimera removal, 199,964 merged reads were used for phylotype calling with DADA2 (Callahan et al., 2016). Finally, 1,204 phylotypes were detected. Singletones and doubletones were removed before diversity analysis.

#### 3.2.1 Preputial microbial diversity does not change after mating

Rarefaction curves showed that the achieved sequencing depth and subsampling size were enough to observe the complete diversity present in the preputial samples. A plateau was reached for richness and evenness metrics (Supplementary Figure S1). No differences were detected for any of the alpha and beta diversity indices calculated.

#### 3.2.2 Aerococcus increases after mating

**Figure 8:**
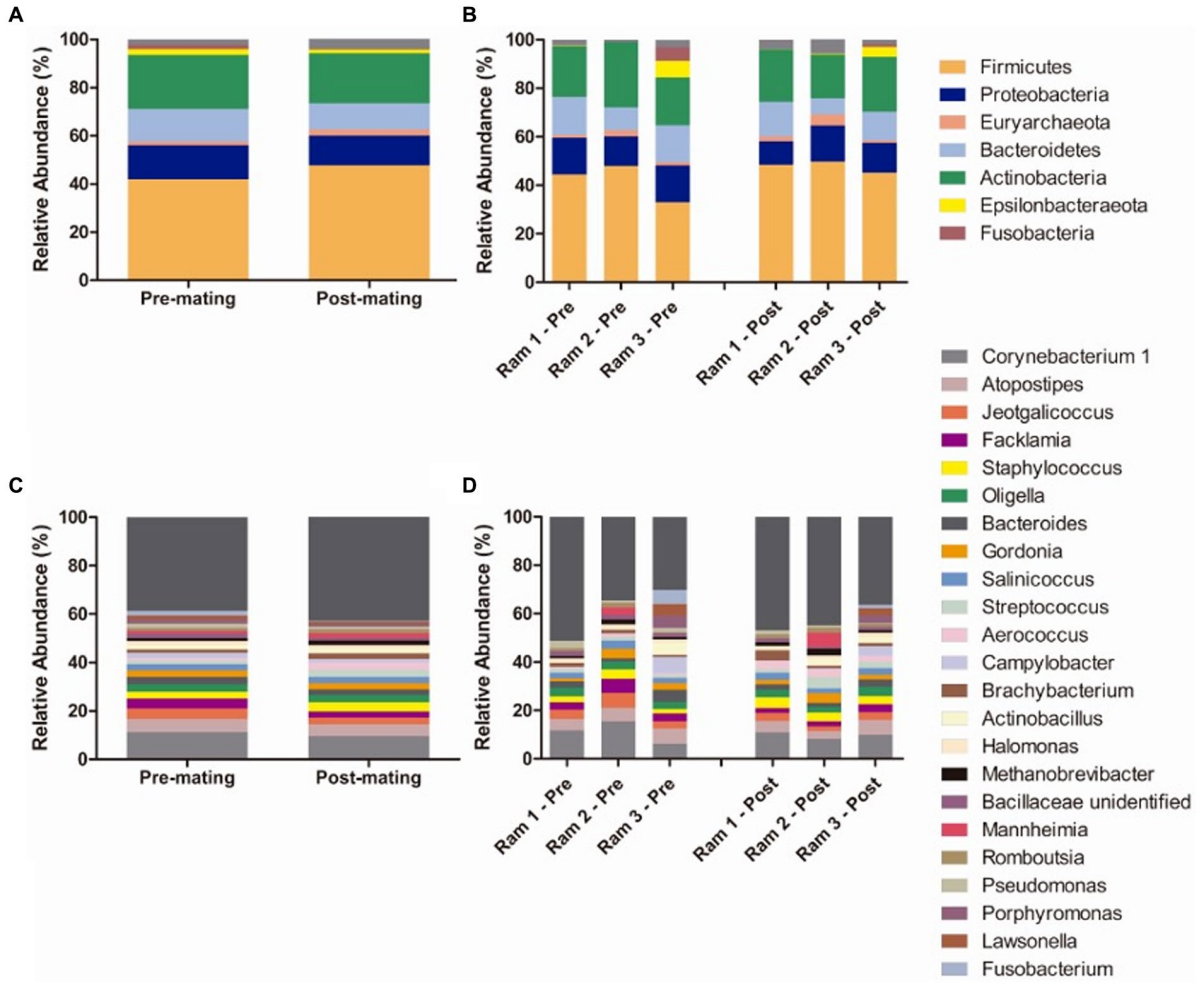
Mean relative abundance of taxa in preputial samples at the phylum level **(A)** and genus level **(C)** and relative abundance of taxa in individual ram samples at phylum **(B)** and genus levels **(D)** at the two sampling points (each column represents an individual sample): pre-mating (Tpre) and post-mating (Tpost). Only taxa with a mean relative abundance >1.5% were plotted.

Sixteen bacterial phyla were detected in preputial samples. The most abundant bacterial phyla were Firmicutes (mean relative abundance Tpre 41.85%, Tpost 47.80%), Actinobacteria (Tpre 26.98%, Tpost 20.74%), Proteobacteria (Tpre 14.16%, Tpost 12.29%), and Bacteroidetes (Tpre 13.53% Tpost 10.90%), followed by Epsilonbacteraeota (Tpre 2.33%, Tpost 1,49%) and Fusobacteria (Tpre 1.88 and 0.42%) (Figure 8). One archeal phylum (Euryarchaeota) was identified (Tpre 1.57%, Tpost 2.53%). No differences in taxonomic composition were observed between Tpre and Tpost samples for the most abundant phyla.

The most abundant genus was *Corynebacterium* 1 (Actinobacteria, Corynebacteriales). Other abundant (>1.5% relative abundance) genera included genus from Firmicutes orders Lactobacillales (*Atopostipes, Aerococcus, Facklamia,* and *Streptococcus*), Bacillales (*Salinococus, Staphylococcus, Jeotgalicoccus* and an unidentified genus from *Bacillaceae* family) and Clostridiales (*Romboutsia*), from Actinobacteria orders Corynebacteriales (*Gordonia* and *Lawsonella*) and Micrococcales (*Brachybacterium*), from Proteobacteria orders Betaproteobacteriales (*Oligella*), Oceanospirillales (*Halomonas*), Pasteurellales (*Actinobacillus* and *Mannheimia*), and Pseudomonales (*Pseudomonas*), from Bacteroidetes order Bacteroidales (*Bacteroides* and *Porphyromonas*), from Epsilonbacteraeota order Campylobacterales (*Campylobacter*), from Fusobacteria order Fusobacteriales (*Fusobacterium*) and from Euryarchaeaota order Methanobacteriales (*Methanobrevibacter*) (Figure 8). *Lactobacillus* was not detected in any of the three rams tested. Differences in the relative abundances of the most abundant genera between Tpre and Tpost samples were only detected for *Aerococcus* (*p* = 0.021), being more abundant in Tpost (3.00%) than Tpre (0.92%) samples.

### 3.3 Vaginal microbiota and preputial microbiota changes related with mating

None of the genera that were significantly increased at Tpost in ram samples were abundant (>1.5%) in ewes at Testrus. No genus significantly decreased at Tpost in rams.

Several genera that were significantly increased in ewes at Tpreg were also abundant (>1.5%) in rams at Tpre. *Actinobacillus*, which was present in the three tested ram Tpre samples (in a range from 4.8 to 0.16% relative abundance) was more abundant in Tpreg_P (21.65%) compared to Testrus (0.008%). *Aerococcus* was more abundant in Tpreg_P (P 1.04%, NP 0.77%) compared to Testrus (P 0.0022%, NP 0%) and was present in all the rams’ Tpre samples (relative abundance ranging from 0.66 to 1.45%). *Ureaplasma*, which was the second most abundant genera both in Tpreg_P and Tpreg_NP, was absent in most individuals at Testrus and was present in only one ram sample at Tpre with 2.28% relative abundance. *Escherichia-Shigella*, which was the most abundant genus in Tpreg_NP, was observed in the same male individual as *Ureaplasma* with 1.9% relative abundance while it was absent in the other two rams.

*Bacteroides* and *Campylobacter* which significantly decreased in ewes in Tpreg_P but did not change significantly in Tpreg_NP, were found to be abundant in Tpre samples. *Bacteroides* was less abundant in Tpreg_P (0.12%) compared to Testrus_P (2.02%), whereas did not vary in NP ewes (Testrus_NP 2.26%, Tpreg_NP 3.40%) and was present in all the rams’ Tpre samples in a relative abundance ranging from 1.31 to 4.91%. Similarly, *Campylobacter* was less abundant in Tpreg_P (1.80%) compared to Testrus_P (10.80%), whereas did not vary in NP ewes (Testrus_NP 5.13%, Tpreg_NP 2.00%) and was present in all rams in a relative abundance ranging from 0.04 to 6.78% at Tpre.

Only *Atopostipes*, among the genera that significantly decreased at Tpreg_NP (0.19%) compared to Testrus_NP (2.17%) did not change significantly in Testrus_P (1.33%) and Tpreg_P (0.93%), was abundant in the rams’ Tpre samples ranging from 4.77 to 6.18% relative abundance.

## 4 Discussion

The present study characterized the vaginal microbiota in nulliparous ewes before the insertion of an intravaginal sponge and two days after the removal of the sponge for estrus synchronization and at pregnancy diagnosis. The foreskin microbiota of the rams before and after natural mating was studied. This is the first time, to the authors’ knowledge, that changes in ovine vaginal microbiota of estrus-synchronized nulliparous ewes using intravaginal sponges and the effect of preputial microbiota, through mating, are evaluated. Nulliparous ewes and rams that had not previously been used for natural mating were used in order to avoid possible bias due to infectious diseases, metabolic disorders related to age of the animals or bacterial vaginal colonization derived from previous mating.

Both Archaea and Bacteria were detected in the vaginal microbiota of ewes, being Bacteria more abundant and showing high diversity. These observations are consistent with previous studies characterizing the vaginal microbiota of other livestock species in a non-diseased state which have identified a higher species diversity compared to humans (Giannattasio-Ferraz et al., 2019; Quereda et al., 2020a). However, differences in the microbiota community structure were observed between Tpreg_P, T0_P and Testrus_P (Supplementary Table S1). Other authors have also reported no diversity nor community structure differences between P group and NP group before the establishment of pregnancy (Serrano et al., 2020; Koester et al., 2021; Reinoso-Peláez et al., 2023). Higher species richness and evenness were observed at T0 compared to Tpreg, both in P and NP groups independently (Figure 1), showing that the bacterial diversity of the vaginal microbiota decreased. Laguardia-Nascimento et al. (2015) reported a decreased bacterial alpha diversity in pregnant cattle compared to non-pregnant ones, indicating a gestational influence on the vaginal microbiota. In this study, the influence of the use of intravaginal sponges should not be ruled out, as it is known to affect the community structure due to competition for nutrients or changes in the vaginal environment, such as fluctuations in vaginal pH (Martinez-Ros et al., 2018). Indeed, a classic long-term estrous synchronization protocol has been linked to fluctuations in the cultivable microbiota present in the vagina of 1 year old ewes (Quereda et al., 2020b). In cows, three microbiome types were linked to post-partum endometritis and all presented poorer vaginal diversity (Miranda-CasoLuengo et al., 2019). A poorer bacterial diversity has also been linked to metritis as these changes may allow the growth of opportunistic pathogenic bacteria (Bicalho et al., 2017). Our results, based on a metagenomic analysis, support these preliminary results. Richness was significantly higher in microbial communities in Testrus_NP compared to Tpreg_NP samples. This could be an indicator of the presence of an abnormal number of bacterial populations in a highly contaminated vagina and therefore a greater risk of the presence of pathogens in it. The risk of vaginosis linked to the use of intravaginal sponges (Gatti et al., 2011; Manes et al., 2013; Martinez-Ros et al., 2018; Quereda et al., 2020b) could lead to favorable ecological conditions for pathogens or opportunistic bacteria colonization (Suárez et al., 2006; Manes et al., 2018).

Several authors reported Firmicutes as the most abundant phylum in ewes (Serrano et al., 2020; Koester et al., 2021; Greenwood et al., 2022; Reinoso-Peláez et al., 2023) and cattle (Laguardia-Nascimento et al., 2015; Bicalho et al., 2017; Giannattasio-Ferraz et al., 2019; Miranda-CasoLuengo et al., 2019; Chen et al., 2020; Quadros et al., 2020). The results of our study are consistent with these previous studies as Firmicutes was always the predominant phylum except in Tpreg_NP. This could evidence a role for Firmicutes in reproductive success from even the beginning of reproductive age. Moreover, as previously suggested, beneficial microorganisms of these groups could be used as vaginal probiotics to improve fertility (Serrano et al., 2020; Quereda et al., 2020b). For example, lactic acid bacteria (LAB) have been proposed to influence the composition of the microbiota in the mammary gland (Toquet et al., 2023) and the reproductive tract (Quereda et al., 2020a) of some ruminants species, mainly by lowering pH through the secretion of lactic acid. Low pH is known to play an important role in the human vagina microbiota by preventing the proliferation of pathogens such as *Mycoplasma genitalium* (Ma et al., 2012; Huppert et al., 2013). Interestingly, in Tpreg_NP, Firmicutes was not the most abundant bacterial phylum. We hypothesize that in the P group the higher proportion of Firmicutes could have avoided the colonization of pathogenic or opportunistic vaginal species. This is in agreement with previous findings showing that LAB *Lactobacillus sakei* and *Weisella koreensis* were decreased in the vaginal microbiota of cows affected by endometritis (Wang et al., 2016). Otero et al. (2006) described several LAB species that could inhibit metritis bovine pathogenic strains of *Actinomyces pyogenes* and *E. coli*, but also *S. aureus* (Otero and Nader-Macías, 2006). Additionally, the *in vitro* capacity of *Lactobacillus* spp. to significantly acidify sperm and cervical mucus and consequently damaging the viability of *Mycoplasma bovis* has been reported in cattle (García-Galán et al., 2020a,b). However, the low abundance of lactobacilli in the ovine vagina, evidenced by a neutral vaginal pH of 6.7 (Swartz et al., 2014), suggests the need not to rule out beneficial effects of other bacterial genera belonging to the Firmicutes bacterial phylum.

Bacteroidetes, Proteobacteria, and Actinobacteria were the other most represented phyla after Firmicutes; this was also observed in previous studies in ruminants (Laguardia-Nascimento et al., 2015; Giannattasio-Ferraz et al., 2019; Chen et al., 2020; Koester et al., 2021). *Porphyromonas* (Bacteroidetes, Bacteriodales) was the most abundant vaginal genus overall and it appears to be a commensal of the ruminant vaginal microbiota as its prevalence has been reported by several authors in cattle (Gonzalez Moreno et al., 2016; Bicalho et al., 2017; Miranda-CasoLuengo et al., 2019; Quereda et al., 2020a) and sheep (Greenwood et al., 2022; Reinoso-Peláez et al., 2023). This genus can also act as an opportunistic pathogen as it was associated with post-partum endometritis in bovine species (Bicalho et al., 2017; Miranda-CasoLuengo et al., 2019). The other two most abundant genera were *Peptoniphilus* (Firmicutes, Clostridiales) and *Anaerococcus* (Firmicutes, Clostridiales). Other authors have detected distinct abundance profiles, for example (Swartz et al., 2014) reported *Aggregatibacter* spp., *Streptobacillus* spp., *Cronobacter* spp., *Phocoenobacter* spp., and *Psychrilyobacter* spp. as the most abundant bacteria in the vaginal microbiota of ewes. Even if our study included mated, pregnant and non-pregnant animals, none of these genera were identified in our study. This suggests that the microbiota is influenced by many factors defined within the herd such as the genotype or breed but also environmental factors like the location of the farm, the diet, and the local farming practices as it has been suggested previously (Toquet et al., 2021). In our study sheep and rams born on the farm during their first natural mating were used, animals from abroad did not enter and no treatments were used in the entire herd, reducing putative variability in the microbiota associated to the mentioned factors. Based on this, the influence on the microbiota of other bacterial communities was reduced in the non-commercial herd used in this study, including effects derived from infections by reproductive pathogenic bacteria linked to diseases such as contagious agalactia, Q fever or listeriosis, which could influence the vaginal or preputial microbiota throughout the life of an animal.

In addition, the ratio Firmicutes/Bacteroidetes was significantly higher in Tpreg_P compared to Tpreg_NP (Figure 4). Bacteroidetes have been previously associated with uterine diseases in cows (Bicalho et al., 2017) and the relationship between Firmicutes and Bacteroidetes has been associated to many pathological conditions and is an indicator of healthy gastrointestinal microbiome in humans (Stojanov et al., 2020). As mentioned previously, in Tpreg_NP, the most abundant phylum was not Firmicutes, but Proteobacteria, which includes enterobacteria, whose proliferation after the use of intravaginal sponges is assumed (Martinez-Ros et al., 2018; Quereda et al., 2020b) has been described a sign of dysbiosis and risk of infection in the human gut microbiota (Shin et al., 2015).

We have observed that the relative abundance of genera *Bacteroides* and *Escherichia-Shigella*, in both groups at Testrus, and *Campylobacter*, in Testrus_P, were increased after the sponge application. Moreover, *Bacteroides* and *Campylobacter*, were significantly lower in Tpreg_P compared to Testrus_P, which could imply a shift to a healthier microbiota, after the sponge induced dysbiosis, that allowed reproductive success. This shift was not observed in NP ewes. These bacteria could have colonized the vaginal microbiota after contamination through the braided silk cords of the sponge that protrude from the vulva and are in contact with feces, urine, and soil. Dysbiosis after the application of intravaginal sponges has been described using culture-dependent methods going from a predominance of Gram-positive bacteria to Gram-negative in sheep (Martins et al., 2009; Manes et al., 2010; Bragança et al., 2017; Ojeda-Hernández et al., 2019). Previously, it was also reported that the increase in *Enterobacteriaceae* in ewes was statistically related to a reduction in diversity (Quereda et al., 2020b). In Tpreg_NP the relative abundance of *Escherichia-Shigella* (and its respective order Enterobacteriales and Phylum Proteobacteria) increased and it became the most abundant genus, whereas in Tpreg_P the abundance decreased. Despite this finding not being statistically significant, it could indicate a possible link between the absence of pregnancy and the dysbiotic state after sponge use. Globally, all the scientific evidence suggests the need to reinforce soil hygiene in sheep flocks with intravaginal sponges, as it is considered as an important management practice to prevent environmental mastitis in dairy cows (Hogan and Smith, 2012).

An interesting finding was that the Tenericutes phylum and its order Mycoplasmatales significantly increased both at Tpreg_P and Tpreg_NP. This group hosts pathogenic, apathogenic, and opportunistic species of *Mycoplasma* spp. in small ruminants (Gómez-Martín et al., 2013). Specifically, *Ureaplasma* sp. USP128 abundance significantly increased at Tpreg becoming the second most represented microorganism in both groups of ewes. One possible explanation for this rise could be the transmission from rams through mating, as one of the males showed the presence of *Ureaplasma* USP128 in its preputial microbiota. Furthermore, *Ureaplasma* was the most abundant genus identified in goat buck semen present in a sperm donor center (Mocé et al., 2022) and among the ten most abundant genera in rams (Serrano et al., 2020). *Ureaplasma* spp. has also been shown to be common in the vaginal microbiota of domestic ruminants (Swartz et al., 2014; Deng et al., 2019; Chen et al., 2020; Quadros et al., 2020; Serrano et al., 2020; Quereda et al., 2020a; Koester et al., 2021; Greenwood et al., 2022). Additionally, Mycoplasmatales are sensitive to drops in the pH of the medium (below 7.4) (Howard and Gourlay, 1978; Gómez-Martín et al., 2015). In this sense, the use of intravaginal devices for 14 days has been reported to increase pH (pH 8 compared to pH 6.8 in sheep without intravaginal devices) (Martinez-Ros et al., 2018). Then, the use of intravaginal sponges for 14 days used in this study could have indirectly favored the colonization of bacterial species such as *Mycoplasma* spp. or *Ureaplasma* spp. by producing pH changes in our study. In this study, *Ureaplasma* spp. may also be linked to pregnancy since it was observed in all P ewes but was only present in two NP ewes. Similar observations were made by (Serrano et al., 2020) where *Ureaplasma diversum* was more abundant in herds with high pregnancy rates. We wondered if the phylum Tenericutes, normally linked to harmful diseases for small ruminants, could play an undervalued role in pregnancy because non-pathogenic bacterial species could occupy the ecological niche of pathogenic species hindering their colonization. It is important to consider that other species of pathogenic Mycoplasmatales with reproductive tropism can colonize the foreskin and vagina of small ruminants, as occurs with the mycoplasmas of contagious agalactia (Gómez-Martín et al., 2013) so that in herds where this infection is not present, apathogenic species of Mycoplasmatales could naturally play a role in the balance of the reproductive microbiota in the absence of other pathogenic bacterial species. The use of progestogen-releasing intravaginal devices causes vaginal conditions that favor the abundance of bacteria included in the order Mycoplasmatales and could be an epidemiological risk factor not previously considered against serious mycoplasmosis of small ruminants such as contagious agalactia. However, the role of bacteria belonging to the phylum Tenericutes and order Mycoplasmatales, such as *Ureaplasma* spp., in small ruminant’s reproduction still needs to be determined.

A noteworthy species was *Actinobacillus seminis* (Proteobacteria, Pasteurellales) which was abundant in both groups at Tpreg but absent at Testrus. This suggests a possible effect of mating on the vaginal microbiota since all rams presented *A. seminis* in the preputial microbiota. Although we could have hypothesized that *A. seminis* was linked to pregnancy given that it was more abundant in Tpreg_P than Tpreg_NP, it is, nevertheless, unlikely since *A. seminis* is a well-known pathogen of the reproductive tract linked with abortions in sheep (Foster et al., 1999) and *Actinobacillus* spp. were reported less abundant in pregnant ewes of commercial sheep farms (Serrano et al., 2020).

In NP ewes, other abundant genera that showed significant differences were: *Alloiococcus* (Firmicutes, Lactobacillales), *Atopostipes*, (Firmicutes, Lactobacillales), and uncultured bacteria W5053 from Family XI (Firmicutes, Clostridiales), which were less abundant at Tpreg than T0 and Testrus. The decrease of these Firmicutes genera could have had a negative impact on the pregnancy outcome. The absence of *Lactobacillus* spp. in 100% of the NP ewes could reflect a possible role of these bacteria in the favorable course of pregnancy or in maintaining bacterial diversity, possibly modulating the pH and avoiding excessively high values. In this sense, their low abundance is linked to the maintenance of ovine vaginal pH and they have been proposed as good candidates for the development of vaginal probiotics that prevent the proliferation of pathogens linked to infertility (Swartz et al., 2014; Serrano et al., 2020; Quereda et al., 2020b; Reinoso-Peláez et al., 2023).

In the rams, the most abundant phyla at Tpre and Tpost were Firmicutes, Actinobacteria, Proteobacteria, and Bacteroidetes which is in agreement with previous reports of the bull and ram preputial microbiota (Serrano et al., 2020; Wickware et al., 2020). One of the most abundant genera we identified in the preputial microbiota included *Corynebacterium, Atopostipes, Salinicoccus*, and *Actinobacillus*. Serrano et al. (2020) also reported these four genera as abundant in the preputial microbiota of rams. *Aerococcus* was the only genus significantly more prevalent after mating. It is interesting to note that *Aerococcus* is also, although not significantly, more prevalent in Tpreg in both groups of ewes, which could imply that both males and females influence the microbiota of one another through mating.

In conclusion, this preliminary study represents the first report of monitoring the vaginal microbiota in nulliparous ewes and the foreskin of rams around the use of intravaginal sponges using metagenomics. Bacterial diversity of the nulliparous ewe’s vaginal microbiota decreased during pregnancy. Firmicutes was the most predominant phylum in both male and female sheep and a decrease in its relative abundance could be linked to infertility. Ewes that became pregnant seemed to have the ability to shift their microbiota to a more balanced state with an important decrease of Bacteroidales, Campylobacterales, and Enterobacterales at the time of gestation, which was not observed in non-pregnant ewes. Other relevant genera involved were *Ureaplasma* and *Actinobacillus* which, although harboring pathogenic species, seemed to have a positive link with the pregnancy. Ram’s preputial microbiota could possibly modulate the vaginal microbiota through natural mating. Interaction between vaginal and preputial microbiota could have detrimental or positive effects not previously contemplated on ovine reproductive physiology around ovine pregnancy. Further studies are needed to reinforce this work and to identify biomarkers and potential probiotic bacteria involved in the gestation outcome.

## Data availability statement

The data presented in the study are deposited in the European Nucleotide Archive (ENA) repository of the European Molecular Biology Laboratory of the European Bioinformatics Institute (https://www.ebi.ac.uk/ena/browser/home), study ID PRJEB63647 (ERP148797).

## Ethics statement

The animal study was approved by University CEU Cardenal Herrera Care and Use Committee for Livestock and by the Spanish Regional Government Generalitat Valenciana (Animal use protocol 2018/VSC/PEA/0183). The study was conducted in accordance with the local legislation and institutional requirements.

## Author contributions

MB, EG-R, and ÁG-M contributed to conception and design of the study. MB, MT, JG, EG-R, JQ, and ÁG-M performed sample collection. PG-T and BC performed metagenomic methodology and statistical analysis. MB, ÁG-M, MT, JQ, JG, and BC wrote sections of the manuscript. All authors contributed to the article and approved the submitted version.

## Footnotes

^1^http://www.R-project.org
