# Supplementary figures and images for: Description of the vaginal microbiota in nulliparous ewes during natural mating and pregnancy: preliminary signs of the male preputial microbiota modulation
Source: Front Microbiol. 2024 Jan 11;14:1224910. doi: 10.3389/fmicb.2023.1224910 (PMC10808482; doi:10.3389/fmicb.2023.1224910)

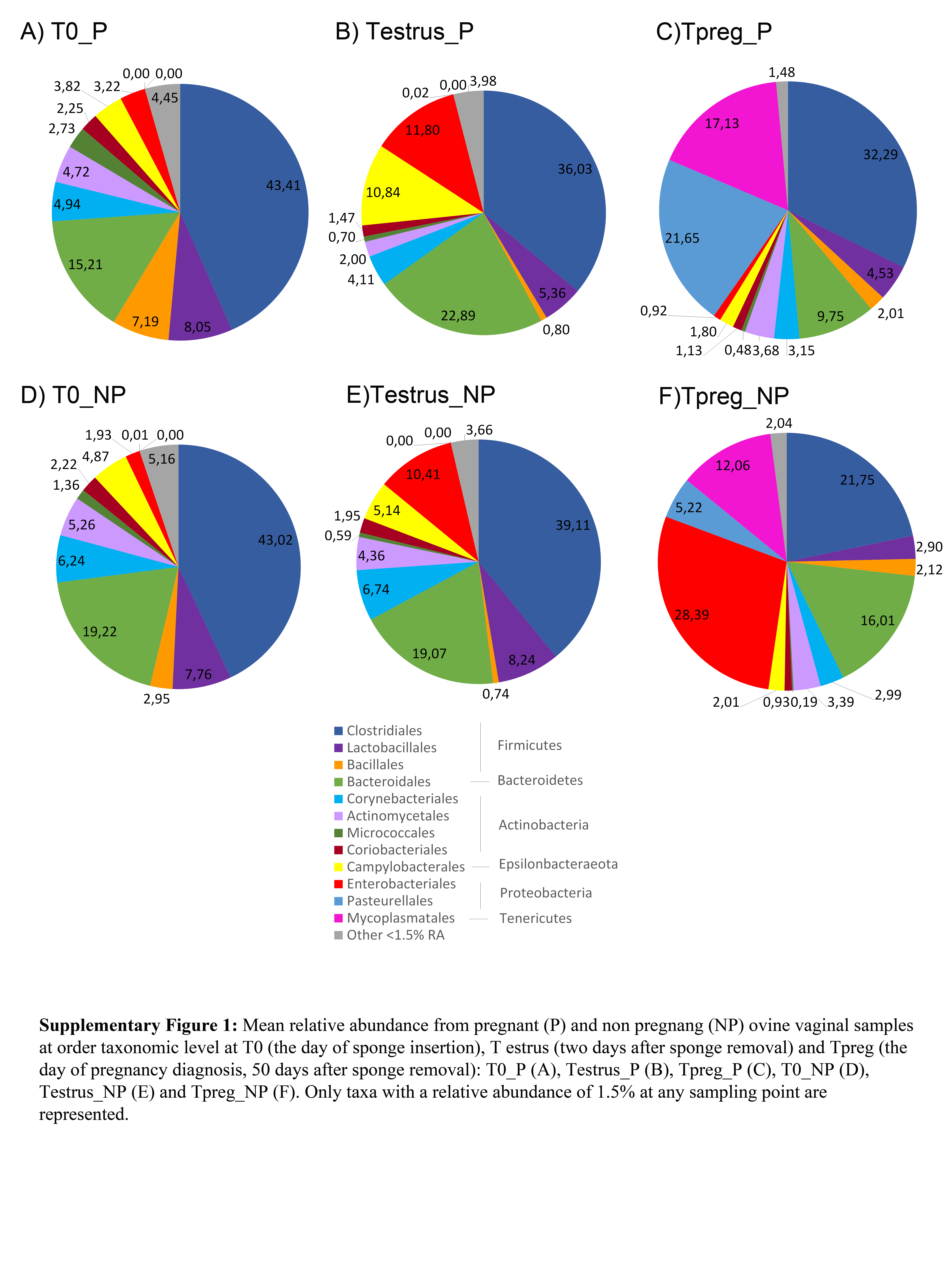

Supplement: Supplementary file 1 [file Image_1.TIF]
