# Supplementary material for: Description of the vaginal microbiota in nulliparous ewes during natural mating and pregnancy: preliminary signs of the male preputial microbiota modulation
Source: Front Microbiol. 2024 Jan 11;14:1224910. doi: 10.3389/fmicb.2023.1224910 (PMC10808482; doi:10.3389/fmicb.2023.1224910)
Supplement: Supplementary file 3 [file Table_1.DOCX]

**Supplementary Table 1.** Median (interquartile interval) of the alpha diversity indices of vaginal microbiota of 10 ewes at the three different sampling points.

| Group | Alpha diversity metric | T0 | Testrus | Tpreg |
| --- | --- | --- | --- | --- |
| P | Richness (Observed ASV´s) | 169,5(128,8)^aA^ | 108 (169,75)^aAB^ | 120 (39)^aB^ |
|  | Evenness (Pielou index) | 0,87 (0,02) ^aA^ | 0,82 (0,17)^aAB^ | 0,84 (0,15)^aB^ |
|  | Richness variance (Shannon index) | 6,44 (0,78)^aA^ | 5,66 (2,23)^aAB^ | 5,9 (1,18)^aB^ |
| NP | Richness (Observed ASV´s) | 250 (96,5)^aA^ | 167 (44)^aA^ | 107,5 (31,3)^aB^ |
|  | Evenness (Pielou index) | 0,87 (0,03) ^aA^ | 0,85 (0,14)^aAB^ | 0,64 (0,37)^aB^ |
|  | Richness variance (Shannon index) | 6,85 (0,76)^aA^ | 6,32 (0,75)^aAB^ | 4,21 (2,72)^aB^ |

P: pregnant, NP: non-pregnant, T0: the day of the sponge insertion, Testrus: two days after sponge removal and Tpreg (the day of pregnancy diagnosis, 50 days after sponge removal), ASV: amplicon sequence variant.

Values in the same column with distinct superscript lowercase letters show significant differences between pregnant and non-pregnant groups within the same sampling time (*P*<0,05). Values in the same line with distinct superscript uppercase letters show significant differences among the experimental times (*P*<0,05). Kruskal-Wallis test followed by Conover’s test with FDR Benjamini-Hochberg correction was added for pairwise comparison.
