# Supplementary material for: Description of the vaginal microbiota in nulliparous ewes during natural mating and pregnancy: preliminary signs of the male preputial microbiota modulation
Source: Front Microbiol. 2024 Jan 11;14:1224910. doi: 10.3389/fmicb.2023.1224910 (PMC10808482; doi:10.3389/fmicb.2023.1224910)
Supplement: Supplementary file 4 [file Table_2.DOCX]

**Supplementary Table 2.** FDR- adjusted *P* values of pairwise comparisons using PERMANOVA analysis for beta diversity indexes matrices.

| Group comparison | Qualitative indexes | | Quantitative indexes | |
| --- | --- | --- | --- | --- |
|  | Jaccard | Unweighted Unifrac | Bray Curtis | Weighted Unifrac |
| T0_P versus T0_NP | 0,764 | 0,476 | 0,867 | 0,707 |
| Testrus_P versus Testrus_NP | 0,753 | 0,413 | 0,729 | 0,707 |
| Tpreg_P versus Tpreg_NP | 0,548 | 0,439 | 0,180 | 0,261 |
| T0_P versus Testrus_P | 0,168 | 0,135 | 0,867 | 0,219 |
| T0_P versus Tpreg_P | 0,015* | 0,030* | 0,030* | 0,020* |
| Testrus_P versus Tpreg_P | 0,100 | 0,133 | 0,030* | 0,020* |
| T0_NP versus Testrus_NP | 0,856 | 0,413 | 0,867 | 0,884 |
| T0_NP versus Tpreg_NP | 0,293 | 0,133 | 0,279 | 0,219 |
| Testrus_NP versus Tpreg_NP | 0,845 | 0,133 | 0,633 | 0,260 |

T0: the day of the sponge insertion, Testrus: two days after sponge removal, Tpreg: the day of pregnancy diagnosis 50 days after sponge removal, P: pregnant ewes, NP: non-pregnant ewes.

Values with * show significant differences.
