# Supplementary material for: Description of the vaginal microbiota in nulliparous ewes during natural mating and pregnancy: preliminary signs of the male preputial microbiota modulation
Source: Front Microbiol. 2024 Jan 11;14:1224910. doi: 10.3389/fmicb.2023.1224910 (PMC10808482; doi:10.3389/fmicb.2023.1224910)
Supplement: Supplementary file 5 [file Table_3.DOCX]

**Supplementary Table 3**. Median relative abundance of genus taxa from vaginal microbiota of 10 ewes that showed significant differences with Kruskal-Wallis testing between the different sampling points in pregnant and non-pregnant ewes.

| Taxa | Group | Median (IQR) Relative Abundance at different sampling times | | *P* value |
| --- | --- | --- | --- | --- |
| *Ureaplasma* | P | T0 | Tpreg | <0,001 |
|  |  | 0 (0) | 13,5 (15,73) |  |
|  | P | Testrus | Tpreg | <0,001 |
|  |  | 0 (0) | 13,5 (15,73) |  |
|  | NP | T0 | Tpreg | 0,011 |
|  |  | 0 (0) | 4,794 (31,37) |  |
|  | NP | Testrus | Tpreg | 0,014 |
|  |  | 0 (0) | 4,794 (31,37) |  |
|  | Tpreg | P ewes | NP ewes | 0,013 |
|  |  | 13,5 (15,73) | 4,794 (31,37) |  |
| *Actinobacillus* | P | T0 | Tpreg | <0,001 |
|  |  | 0 (0) | 16,31 (28,913) |  |
|  | P | Testrus | Tpreg | <0,001 |
|  |  | 0 (0,013) | 16,31 (28,913) |  |
|  | Tpreg | P ewes | NP ewes | 0,002 |
|  |  | 16,31 (28,913) | 0 (15,67) |  |
| *Murdochiella* | P | T0 | Testrus | 0,019 |
|  |  | 4,12 (2,789) | 0,832 (2,031) |  |
|  | P | Testrus | Tpreg | 0,003 |
|  |  | 0,832 (2,031) | 5,359 (5,201) |  |
| *Campylobacter* | P | Testrus | Tpreg | 0,012 |
|  |  | 8,938 (11,566) | 2,059 (1,989) |  |
| *Alloiococcus* | NP | T0 | Tpreg | 0,024 |
|  |  | 2,282 (1,39) | 0,1153 (0,199) |  |
|  | NP | Testrus | Tpreg | 0,024 |
|  |  | 1,685 (2,182) | 0,1153 (0,199) |  |
| *Atopostipes* | NP | T0 | Tpreg | 0,028 |
|  |  | 2,325 (3,532) | 0,204 (0,299) |  |
|  | NP | Testrus | Tpreg | 0,034 |
|  |  | 2,898 (3,088) | 0,204 (0,299) |  |
| uncultured bacteria W5053 from Family XI | NP | T0 | Tpreg | 0,015 |
|  |  | 2,425 (0,85) | 0,3065 (0,2461) |  |
|  | NP | Testrus | Tpreg | 0,015 |
|  |  | 1,851 (2,691) | 0,3065 (0,2461) |  |

P: pregnant ewes, NP: non-pregnant ewes, T0: the day of the sponge insertion, Testrus: two days after sponge removal, Tpreg: the day of pregnancy diagnosis 50 days after sponge removal.
